# Engaging the spikes: heparan sulfate facilitates SARS-CoV-2 spike protein binding to ACE2 and potentiates viral infection

**DOI:** 10.1038/s41392-021-00470-1

**Published:** 2021-01-29

**Authors:** Rajkumar Singh Kalra, Ramesh Kandimalla

**Affiliations:** 1 AIST-INDIA DAILAB, National Institute of Advanced Industrial Science & Technology (AIST), Higashi 1-1-1, Tsukuba, Japan; 2 Applied Biology, CSIR-Indian Institute of Chemical Technology (IICT), Uppal Road, Tarnaka, Hyderabad, Telangana State India; 3 Department of Biochemistry, Kakatiya Medical College, Warangal, Telangana 506004 India; 4 Present Address: Immune Signal Unit, Okinawa Institute of Science and Technology Graduate University, 1919-1 Tancha, Onna-son, Okinawa 904-0495 Japan

**Keywords:** Genetic engineering, Predictive markers

In a recent report published in *Cell*, Clausen et al.^1^ reveal that heparan sulfate (HS) functions as a necessary cofactor for SARS-CoV-2 binding to the ACE2 at the host cell membrane. Molecular analysis identified that HS interacts with the receptor-binding domain (RBD) at the S1 subunit of the SARS-CoV-2 trimeric S-protein, which facilitates the opening of S-protein conformation for ACE2 binding.^1^

Coronavirus disease (COVID-19) has emerged as a major pandemic of the modern age. Ongoing COVID-19, caused by severe acute respiratory syndrome coronavirus 2 (SARS-CoV-2), has overly strained the global healthcare system with overwhelmed infectivity and incessant mortalities. Consorted efforts are globally being made to understand the mechanism for SARS-CoV-2 infection in the host to clinically intervene in the SARS-CoV-2 infection and its ever-evolving spread.

**Fig. 1:**
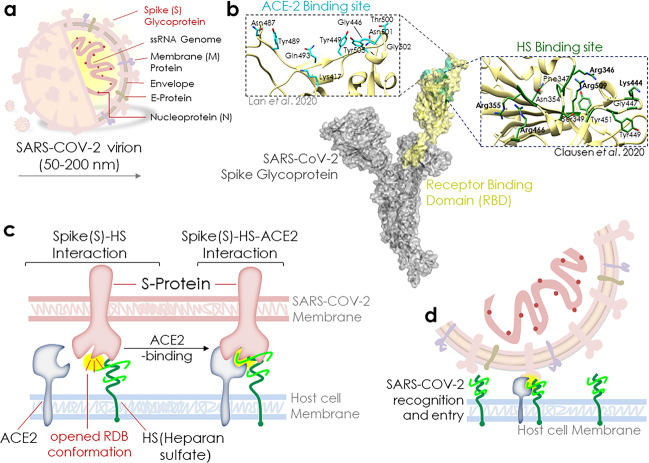
Heparan sulfate enhances SARS-CoV-2 spike protein binding to ACE2 and potentiates viral infection. **a** SARS-CoV-2 virion and spike (S) glycoprotein. **b** A 1273-amino acid-long SARS-CoV-2 S glycoprotein. Receptor-binding domain (RBD) that interacts with the host cell surface ACE2 receptor is exhibited here in yellow. A network of hydrophilic (13H bonds and two salt bridges—shown in cyan) interactions contribute to this binding of ACE2 to RBD (Lan et al. 2020), whereas a putative HS-binding site is shown just adjacent to the ACE2-binding site in RBD. The key residues suggested to involve in the interaction are shown in green on the right of RBD. **c**. Schematic diagram showing HS-led open conformation of RBD and subsequent spike(S)-HS-ACE2 interaction. **d** Schematic model depicting SARS-COV-2 recognition and entry

Shedding light on the mechanism involved in the reception of SARS-CoV-2 at the cell surface and its facilitation before its binding to the specific receptor, viz., ACE2 (angiotensin-converting enzyme 2) can significantly contribute to the present understanding of SARS-CoV-2 infection and spread. SARS-CoV-2 virion (50–200 nm) possesses trimeric spike (S) glycoprotein at the surface that is central to SARS-CoV-2’s host cell binding and infection (Fig. 1a, b). Recent report by Clausen et al. sheds light on the key role of HS in the SARS-CoV-2 binding to the ACE2 at the cell membrane.^1^ They revealed that HS interacts with residues, adjacent to the ACE2-binding site at the RBD of the S1 subunit of the SARS-CoV-2 trimeric S-protein, and this event, in turn, promotes the opening of S-protein conformation for ACE2 binding and thereby potentiates viral infection (Fig. 1b).

HS is a glycocalyx polysaccharide that possesses a high negative charge and frequently allies with membrane or extracellular matrix proteoglycans.^1^ HS position makes it a primary site for virus and other pathogen engagement at the cell membrane, much before its access to a specific receptor and host cell entry attempt. Clausen et al.^1^ have discovered that HS has a vital role to play in the binding of SARS-CoV-2 S-protein to ACE2. HS binding to S-protein was suggested to alter the latter structure from a “close → open” RBD conformation that favors and concurrently potentiates ACE2 binding. Computational analysis revealed that negatively charged HS strongly interacts with positively charged Arg346, Arg355, Lys444, Arg466, and probably Arg509 amino acid residues of the S-protein RBD (Fig. 1b, residues in bold). While a few other residues, viz., Phe347, Ser349, Asn354, Gly447, Tyr449, and Tyr451, were suggested to play an auxiliary role in the binding by coordinating the H-bond and hydrophobic interactions (Fig. 1b, residues in the plain font). Lan et al.^2^ earlier showed that the Asn487, Tyr489, Gln493, Tyr449, Gly446, Thr500, Asn501, Gly502, and Lys417 residues in the S-protein RBD are engaged in ACE2 recognition and binding (Fig. 1b, inset-ACE2-binding site). Hence, it corroborated that the adjacent loci of RBD can bind to HS and ACE2 simultaneously, yet in a cooperative manner. Given the early RBD to HS binding, HS facilitates the initial SARS-CoV-2 virion docking to the host cell, yet the viral entry requires its subsequent transfer to a protein receptor. These results emphasized the role of HS as a “virus collector”, given its direct engagement with virus particles at the cell membrane and subsequently facilitating the open RBD conformation (Fig. 1c, d). It further explains that how besides the ACE2 expression level, HS greatly exhibits the tissue tropism and associates with the altered susceptibility of patients.

At the evolutionary side, RBD of S-protein shows 73% similarity between SARS-CoV-1 and SARS-CoV-2. Of note, most of the positively charged residues at the RBD surface are conserved between them, except the Lys444 in SARS-CoV-2, which is a Thr in SARS-CoV-1. While, in other conserved residues, Asn354 existed in SARS-CoV-2 that is originally a negatively charged Glu in SARS-CoV-1. Clausen et al.^1^ suggested that these 2 substitutions of Thr → Lys444 and Glu → Asn354 from SARS-CoV-1 to SARS-CoV-2 potentially evolved the electropositive affinity of RBD to HS that was reflected in their enhanced interaction in SARS-CoV-2. In agreement this, SARS-CoV-2-recombinant RBD protein exhibited a stronger affinity with heparin-bovine serum albumin, as compared to SARS-CoV-1. A recent report by Kim et al.^3^ also corroborated this finding by showing that immobilized heparin binds more strongly to the SARS-CoV-2 monomeric and trimeric S-protein than that of the SARS-CoV and MERS-CoV.

Lang et al.^4^ earlier showed that SARS-CoV-1 besides its entry receptors ACE2 and the transmembrane protease serine 2 (TMPRSS2) also establishes contact with HS at the cell surface. Here, Clausen et al.^1^ demonstrated that how a stretch of positively charged residues at the RBD domain establishes a strong interaction with HS, thereby, affirming how the electropositive affinity of RBD evolved from SARS-CoV-1 to SARS-CoV-2. Moreover, it revealed that HS and heparin compete for the SARS-CoV-2 S-protein RBD binding, while an inhibitory effect of heparin on pseudovirus and authentic SARS-CoV-2 infectivity was observed.^1^ To order to assess the specificity of HS interaction with SARS-CoV-2 S-protein, a study by Kim et al.^3^ analyzed the competitive binding kinetics of S-protein with another glycosaminoglycan (GAGs), including different chondroitin sulfates, dermatan, and keratan sulfates, revealing weak or no binding activities of these GAGs. It, therefore, suggested that among different GAGs, HS interaction with SARS-CoV-2 protein is highly specific. Also, they showed that the sulfate groups within heparin have a critical impact on its interaction with SARS-CoV-2 S-protein as the chemically modified heparins lacked a competitive binding to the latter.^3^ While analyzing the antiviral properties of heparin-related GAGs and sulfated polysaccharides in vitro, Kwon et al.^5^ further reaffirmed specific sulfonated heparin binding to Sprotein and suggested their therapeutic utility for COVID-19 patients in the clinics. Taken together, Clausen et al.^1^ and these reports underlined the therapeutic potential of heparins (unfractionated heparin (UFH), non-anticoagulant heparin, and heparin lyases) by competing/targeting the pseudotyped virus or authentic SARS-CoV-2 interaction with HS to control SARS-CoV-2 infection and spread.^3–5^

Thrombotic complications are unrealistically frequent in COVID-19 patients. These include cardiac thrombosis, vascular or disseminated intravascular microthromboses, venous thromboembolism, and stroke, limb/mesenteric ischemia and therefore frequently treated with therapeutic UFH or low-molecular-weight heparin (LMWH). Here, Clausen et al.^1^ further identified that the anticoagulant function of heparin (lacking in HS) is independent of its viral-inhibitory activity. These results corroborated therapeutics usage of UFH and LMWH against SARS-CoV-2 infection, essentially by inhibiting the S-protein binding to the cell.^1,3–5^ Although findings by Clausen et al. provided insights into the role of HS in SARS-CoV-2 infection, further research is warranted to exercise its therapeutic potential in clinics. Also, these results stress the necessity to delineate the antiviral or anticoagulant efficacy and dosage of heparin and non-anticoagulant heparins in the clinic against COVID-19.
